# A binational USA-Mexico COVID-19 vaccine clinic: A novel model for cross-border collaboration in health crisis

**DOI:** 10.7189/jogh.12.03012

**Published:** 2022-05-30

**Authors:** Shira Abeles, Linda Hill, Erika Melissa Machado Peña, Lydia Ikeda

**Affiliations:** 1 Division of Infectious Diseases and Global Public Health, Department of Medicine, University of California San Diego Health, San Diego, California, USA; 2 Department of Family Medicine and Public Health, University of California San Diego Health, San Diego, California, USA; 3 Jurisdiction of Tijuana Health Services, Baja California, Mexico; 4 University of California San Diego Health Physician Group, San Diego, California, USA

San Diego, California and Tijuana, Baja California are divided by an international border but inhabited by a shared population. The COVID-19 pandemic highlighted how these cities are not only geographically and socioeconomically tied, but epidemiologically linked as well. The cities differ, however, in terms of resources, and this was acutely highlighted by their access to COVID-19 vaccines. As of May 2021, over 60% of San Diegans had been vaccinated with a COVID-19 vaccine and supply had exceeded demand, while access to effective vaccines in Mexico remained extremely limited. As of May 2021, Baja California was vaccinating the general public aged 50 and above but was struggling with vaccine accessibility and the reliability of the vaccination process.

Maquiladoras are manufacturing plants in Mexico that are foreign-owned. They process or assemble imported components for export and are permitted to temporarily import materials into Mexico on a duty-free basis. There are about 1000 maquiladoras in Baja California, of which approximately half are US-owned. These plants employ roughly 400 000 essential workers and operate largely under US oversight. These essential workers continued to work in person throughout the pandemic, facing an increased risk of infection. In essence, they were working in US-based companies but were not eligible for vaccination when essential workers were offered vaccines in the US.

Leaders of some of these maquiladoras had worked for months to obtain access to vaccines for essential workers in Mexico, but the process was fraught with challenges. The Mexican government had a limited supply, so some individuals and businesses sought to purchase vaccines independently in international markets, needing to navigate and avoid potential fraudulent arrangements. After various forays to attempt to acquire vaccine, the maquiladora local leaders communicated with the Mexican Consulate in San Diego regarding this effort. The Mexican Consulate in San Diego and the University of California, San Diego (UCSD) had established close ties since the onset of the pandemic, working in the Cali-Baja region on COVID-19 prevention and mitigation, including an epidemiologic study of COVID-19 incidence and prevalence in Baja California. The binational effort to vaccinate across the border ensued. We describe the process in which these parties from San Diego and Tijuana worked together and arranged for a binational vaccine clinic to vaccinate essential workers in maquiladoras in Mexico.

UCSD Health had been operating a mobile vaccination unit (MVU) as part of its mission to vaccinate the region and extend COVID-19 vaccines to populations who were most impacted by COVID-19 and faced barriers accessing them. This mobile unit had been going into the communities to vaccinate since the beginning of March 2021. The connection was made between the MVU and the mission of the maquiladoras. UCSD Health involved San Diego County Public Health, which in turn consulted with the Centers for Disease Control and, ultimately, the White House to gain approvals for this binational vaccine campaign. All consulted organizations approved this effort, recognizing the disparity among nations, and the importance of ongoing global vaccine efforts to accelerate world recovery from the pandemic. After all of the proper approvals were secured, multiple meetings were held with UCSD Health, the Mexican Consulate, the maquiladora owners participating in the initial pilot, Customs and Border Protection from the San Diego / Tijuana border, and the County of San Diego.

The Consulate worked with proper authorities to secure a location for the vaccine clinic. A neutral zone was established close to the San Ysidro, California border crossing station. The final site was literally on the border, structured to allow vaccines to be given without formal entry of the vaccinees into the US, obviating the need for formal admittance to the US. The planning included multiple site visits to design and optimize the flow for the vaccination clinic, ensuring that operational resources (such as electricity and internet access) were adequate. Furthermore, in anticipation of media interest, the site was set up with care to ensure patient privacy from media cameras. The initial pilot targeted to vaccinate 1500 workers per day over seven days to reach 10 000. The Janssen (Johnson & Johnson) COVID-19 vaccine was preferred by participating sites given the single-dose regimen simplifying the complex logistics of binational vaccination administration.

Patient educational resources were provided to medical leadership from Mexico in order to make information available to essential workers being offered the vaccine. This included the Spanish version of the vaccine information sheet from the FDA [1], formatted into a singular page, as well as a one-page information sheet on the J&J vaccine produced by the California Department of Public Health (CDPH) [2]. Essential workers then opted into the vaccination process so that a schedule could be made to transport vaccine recipients to the vaccine site, ensure the appropriate number of doses of vaccine were delivered to the vaccine clinic site, and assign the appropriate number of bilingual staff to the vaccine clinic for each day of operations.

**Figure:**
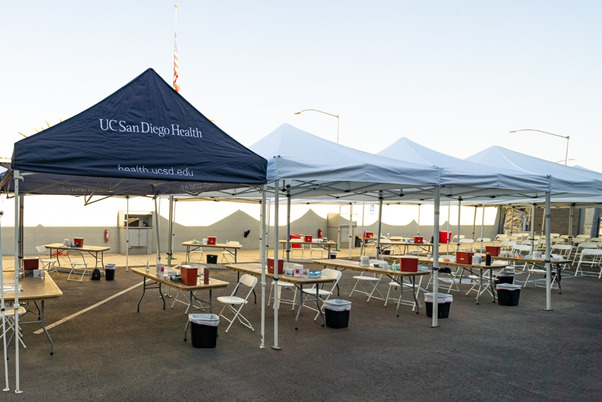
Photo: From Kyle Dykes, University of California San Diego Health (used with permission).

Participating maquiladoras arranged bus transportation for essential workers to the vaccine site. Essential workers were instructed to eat and drink prior to getting to the site to decrease risk of vasovagal response to vaccination. Once on site, persons were verified as recorded participants upon getting off the bus, and then walked to a registration site. Minimum registration requirements had already been established as a key component in UCSD Health’s mobile vaccine outreach operations, to help booster vaccinations in vaccine-hesitant communities so that some of the typical identifiers, such as a social security number, were not required. Persons presenting for vaccination were registered into the UCSD Health system in this expedited fashion. To optimize efficiency, in addition to registration, pertinent medical history was documented, the vaccine appointment was scheduled, basic screening questions (including allergies to vaccine components and history of anaphylaxis) were performed and documented, and vaccination cards were filled out, all prior to proceeding to vaccination tables.

After registration, patients were directed to a vaccination station where bilingual vaccinators reviewed information regarding vaccine efficacy and possible side effects of the vaccine and allowed for any questions, utilizing the CDPH information sheet that was available in Spanish [2] and English [3]. Physician oversight was provided for any additional questions not able to be answered by experienced nurses on site.

After vaccination, patients proceeded to a shaded outdoor observation area for 15-30 minutes prior to returning in groups to the buses and proceeding back to the worksite. If there were emergencies, the UCSD medical staff was present to triage and manage any immediate reactions or vasovagal events. An emergency kit containing diphenhydramine and epi-pens was located on-site. There was an ambulance stationed on the Mexican side of the border in case of emergency. In case of any unanticipated delay with the emergency vehicle in Tijuana, a backup would be to utilize 911 services. Persons vaccinated were entered into the San Diego County and California immunization registries. Names of persons vaccinated were also given to the Baja California Health Secretary for documentation in Mexico.

Approximately 1200-1800 vaccines were administered per day at the vaccine site from May 24, 2021, to June 29, 2021. There were no severe reactions, and no need for emergency services. Once the press release and media advisory were disclosed, multiple binational factories contacted UCSD Health to request inclusion in the vaccination project, or guidance in replicating this effort at other sites along the border in similar binational communities. Requests continue, which only highlights the disparity and hunger for the vaccine for our southern neighbours. Our binational vaccine clinic continued until the vaccine site was no longer available. A total of 26 464 essential workers from maquiladoras were safely administered COVID-19 vaccines.

No pathogen, especially one as infectious as COVID-19, respects man-made borders. The COVID-19 vaccines are highly efficacious, and the pandemic will not be over until the global population has robust immunity to the virus. The faster we vaccinate, the fewer the opportunities for the virus to replicate and mutate into further variants that could place our societies again at risk of repeat surges and associated morbidity, mortality, and large-scale suffering. This binational partnership is the first effort of its kind that we know of along the USA-Mexico border. Expanding access to essential workers in the region improved the resilience of the region’s border by protecting the health of communities spanning both sides of the border against COVID-19. The collaboration likewise strengthened relationships within the Baja region. Our hope is that this binational collaboration can be replicated along the border to foster faster vaccination of our global community and allow for improved binational relationships to improve further health of our communities. This program is a model of successful binational collaboration, involving multiple governmental and non-governmental agencies, which can be applied to other public health crises.
